# The Complexity of Piroplasms Life Cycles

**DOI:** 10.3389/fcimb.2018.00248

**Published:** 2018-07-23

**Authors:** Marie Jalovecka, Ondrej Hajdusek, Daniel Sojka, Petr Kopacek, Laurence Malandrin

**Affiliations:** ^1^BIOEPAR, INRA, Oniris, Université Bretagne Loire, Nantes, France; ^2^Institute of Parasitology, Biology Centre of the Czech Academy of Sciences, České Budějovice, Czechia; ^3^Faculty of Science, University of South Bohemia, České Budějovice, Czechia

**Keywords:** piroplasms, *Babesia*, *Theileria*, developmental cycle, merogony, gamogony, sporogony

## Abstract

Although apicomplexan parasites of the group Piroplasmida represent commonly identified global risks to both animals and humans, detailed knowledge of their life cycles is surprisingly limited. Such a discrepancy results from incomplete literature reports, nomenclature disunity and recently, from large numbers of newly described species. This review intends to collate and summarize current knowledge with respect to piroplasm phylogeny. Moreover, it provides a comprehensive view of developmental events of *Babesia, Theileria*, and *Cytauxzoon* representative species, focusing on uniform consensus of three consecutive phases: (i) schizogony and merogony, asexual multiplication in blood cells of the vertebrate host; (ii) gamogony, sexual reproduction inside the tick midgut, later followed by invasion of kinetes into the tick internal tissues; and (iii) sporogony, asexual proliferation in tick salivary glands resulting in the formation of sporozoites. However, many fundamental differences in this general consensus occur and this review identifies variables that should be analyzed prior to further development of specific anti-piroplasm strategies, including the attractive targeting of life cycle stages of *Babesia* or *Theileria* tick vectors.

## Introduction

The group Piroplasmida received its name after its pear-shaped (pyriform) intra-erythrocytic stages and refers to intracellular parasites transmitted exclusively by hard ticks (Ixodidae) (Mehlhorn and Schein, 1993; Votypka et al., 2017). Piroplasms belong to the most common group of mammalian blood parasites and their impact economically, as well as on veterinary and medical care, is significant. Due to the worldwide distribution of tick vectors, babesiosis is the most common blood disease of free living animals (Homer et al., 2000; Hunfeld et al., 2008) and is considered an emergent zoonosis of humans (Homer et al., 2000; Kjemtrup and Conrad, 2000; Zintl et al., 2003; Hunfeld et al., 2008; Leiby, 2011). From the veterinary point of view, great attention is paid to bovine babesiosis, which is associated with mortalities, abortions, decreased meat, and milk production, but despite permanent epidemiological surveillance, most of the 1–2 billion cattle worldwide are still exposed to babesiosis and outbreaks occur frequently (Bock et al., 2004; Gohil et al., 2013). Humans are not natural hosts for any species of *Babesia* but serve as accidental hosts (reviewed in e.g., Yabsley and Shock, 2013). Despite this fact, the incidence of human babesiosis is on the rise and clinical cases have been reported recently from many countries worldwide (reviewed in e.g., Yabsley and Shock, 2013; Vannier et al., 2015).

Taxonomic classification places Piroplasmida species in the phylum Apicomplexa, as close relatives of the malarial disease agents, *Plasmodium* parasites (e.g., Burki et al., 2009; Janouskovec et al., 2010; Arisue and Hashimoto, 2014; Schreeg et al., 2016). Based on multi-gene analyses, the order Piroplasmida includes three genera, *Babesia, Theileria*, and *Cytauxzoon*. Piroplasms share many morphological and developmental features such as apical complex organelles, merogony (asexual multiplication) within erythrocytes of vertebrate hosts and sexual multiplication followed by sporozoite formation in invertebrate vectors, ticks (Homer et al., 2000). There are five evolutionary lineages recognized in the order Piroplasmida (Schreeg et al., 2016). All these lineages differ in particular developmental features and possess unique adaptations (Table 1). The lifecycle of piroplasms is considered as only partially elucidated. There are many inconsistencies about crucial developmental events of piroplasms, and relevant information are spread throughout many publications. The recent nomenclature changes and redescription of many species has also contributed to these misconceptions (e.g., Mehlhorn and Schein, 1998; Malandrin et al., 2010; Baneth et al., 2015).

**Table 1:** Summarizing overview of characteristic life cycle events of five evolutionary lineages of the order Piroplasmida (based on Schreeg et al., 2016).

	***Babesia* sensu stricto**	***Theileria* sensu stricto**	***Theileria equi***	**Western *Babesia* group**	***Babesia microti* group**
Reduced apical complex organelles	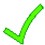	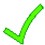	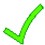	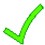	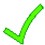 ^**a**^
Schizogony in nucleated blood cells	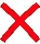 ^**b**^	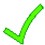	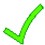	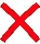 ^**b**^	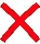 ^**b**^
Neoplastic transformation of the nucleated blood cells	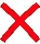	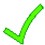 ^**c**^	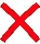	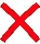	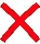
Merogony in red blood cells	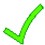 ^**d**^	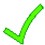 ^**d**^	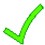 ^**d**^	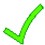 ^**d**^	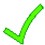 ^**d**^
Motile sporozoites	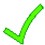	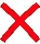 ^**e**^	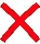 ^**c**^	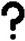	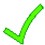
Parasitophorous vacuole formation	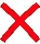	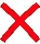	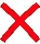	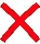	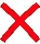
Piriform shape of merozoites	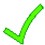	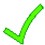	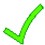	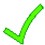 ^**f**^	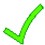 ^**f**^
Gametocytes in the host bloodstream	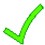 ^**g**^	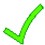 ^**g**^	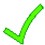 ^**g**^	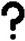	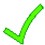 ^**g**^
Strahlenkörpers/spiky-rayed gametes	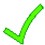 ^**h**^	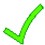	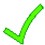	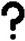	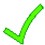 ^**h**^
Macro- and micro-gametes differentiation	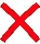	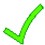 ^**i**^	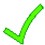 ^**i**^	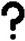	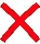
Zygote formation	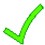 ^**j**^	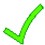 ^**j**^	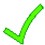 ^**j**^	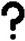	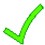 ^**j**^
Primary kinetogenesis in epithelial cells	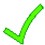	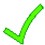	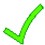	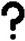	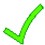
Invasion of primary kinetes directly to salivary glands	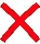	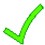	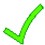	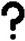	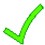 ^**k**^
Secondary kinetogenesis in tick tissues	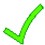	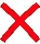	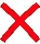	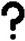	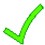
Kinetes invasion into ovaries, transovarial transmission	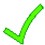	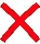	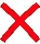	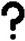	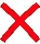
Sporogony in tick salivary glands	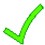	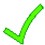	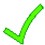 ^**l**^	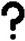	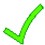
Hypertrophy of infected acini cells	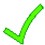 ^**m**^	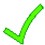	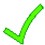	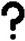	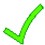
Cytomeres formation during sporoblast maturation	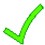	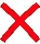	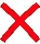	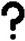	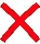
Polar rings formation in sporozoites	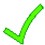	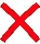	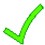	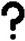	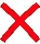
Asynchronous sporozoites release by budding process	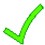 ^**n**^	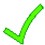	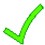	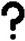	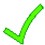

*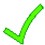, present; 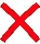, absent; 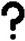, not elucidated yet; a, more reduced compared to other piroplasms; b, absence of schizonts has not been convincingly demonstrated for several species in these groups yet; c, neoplastic transformation of the nucleated host blood cells has been reported for only few species; d, merogony is not synchronous, thus trophozoites and merozoites occur in the bloodstream simultaneously; e, sporozoites do not require apical-end first orientation to internalize into the host cell; f, more frequently ovoid and/or polymorphic shape; g, undistinguishable by light microscopy; h, two gametes (Strahlenkörper) populations which are undistinguishable by light microscopy; I, formation of macrogametes (ovoid shape) and microgametes (Strahlenkörper, spiky-rayed shape); j, zygote is motile and penetrates the peritrophic matrix to internalize into midgut epithelial cell; k, B. microti is able to directly invade tick salivary glands where the secondary kinetogenesis can take place; l, although generally the sporozoites maturation starts after attachment of molted tick stage onto host, T. equi sporozoites can mature prior tick molting; m, hypertrophy of infected acini cells was confirmed for some species (e.g., B. bovis and B. canis) but excluded for some other species (e.g., B. ovis and B. cabalii); n, apart from B. canis, where sporozoite differentiation was described as a result of successive binary fissions. The table is composed based on references provided in the text*.

In this review we provide a comprehensive overview of piroplasm lifecycle events, proposing uniform consensus and stressing unique developmental adaptations with respect to evolutionary lineages.

## Schizogony and merogony: asexual multiplication in blood cells of the vertebrate host

**Figure 1:**
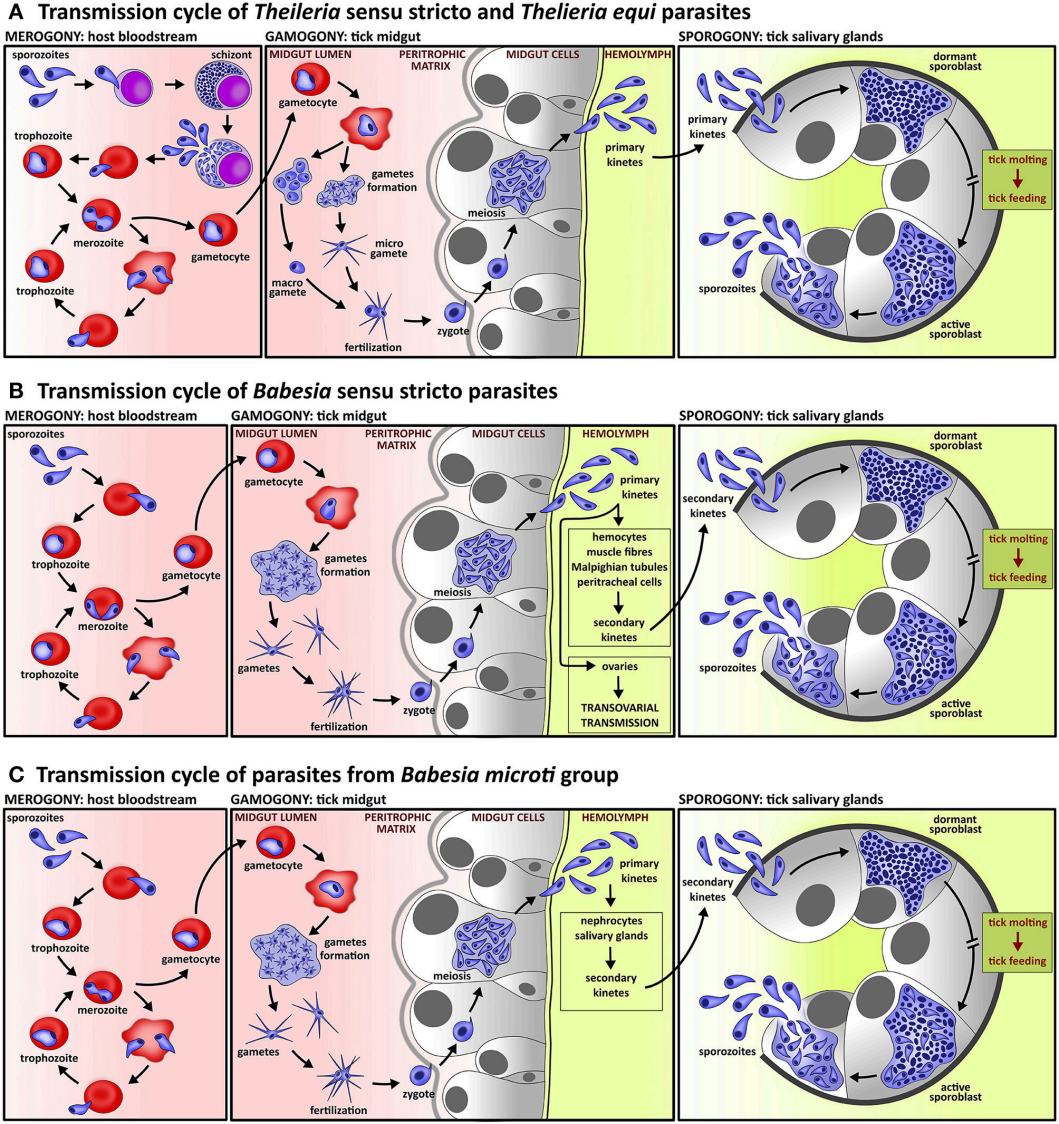
Lifecycle of piroplasms. **(A)** Lifecycle of species of *Theileria* sensu stricto lineage and *Theileria equi* includes intra-leucocytic schizogony prior to intra-erythrocytic merogony; schizogony is often accompanied by neoplastic transformation of host leucocytes. In contrast to *Babesia* species, the gametes of *Theileria* parasites form two morphologically distinguishable cell types, micro- and macro-gametes. Kinetes, which are produced in tick midgut cells, migrate directly to tick salivary glands where sporogony takes place. **(B)** Lifecycle of *Babesia* sensu stricto species comprise exclusively intra-erythrocytic asexual multiplication. Fertilization, which takes place in the tick midgut, is provoked by fusion of two morphologically indistinguishable gametes. The primary kinetes released from tick midgut cells invade various tick tissues, where secondary kinetes are produced. These then invade the tick salivary glands and undergo sporogony. All species of the lineage *Babesia* sensu stricto exploits transovarial transmission, a unique strategy of parasite invasion into ovarian cells by primary kinetes which results in *Babesia*-infected tick eggs and subsequent larvae. **(C)** The life cycle of *Babesia microti* group, the basal lineage of piroplasms, differs from species of the *Babesia* sensu stricto lineage by the lack of transovarial transmission.

All parasites of the group Piroplasmida reproduce asexually inside the blood cells of the vertebrate host (Figure 1, Table 1). The host infection is initiated by the invasion of sporozoites, transmitted through saliva secretion during the tick bite. The blood cells targeted by sporozoites differ according to the species of piroplasm (Shaw, 2003; Lobo et al., 2012). *Theileria* parasites are characterized by schizogony (Box 1) in nucleated blood cells—monocytes and lymphocytes—prior to red blood cell invasion (Schein et al., 1981; Moltmann et al., 1983a; Conrad et al., 1985; Webster et al., 1985; Dobbelaere and Heussler, 1999; Dobbelaere and Rottenberg, 2003; Shaw, 2003). *Babesia* parasites are believed to multiply exclusively in erythrocytes; so far a schizogony has never been convincingly confirmed (Mehlhorn and Schein, 1993; Lobo et al., 2012; Schreeg et al., 2016).

Box 1 **Subsequent phases of piroplasm development.**

**Schizogony**. A process of asexual multiplication in nucleated blood cells (leukocytes) is typical only for two evolutionary lineages of piroplasms, *Theileria* sensu stricto and *Theileria equi*. Schizogony starts after sporozoite internalization into leukocytes and results in merozoite production, which further multiply by merogony. Schizogony can lead to neoplastic transformation of the nucleated host cells, which then proliferate indefinitely. **Merogony**. A process of asexual division in the red blood cell starts either with sporozoite (*Babesia species*) or merozoite (*Theileria* species) invasion of red blood cells. The internalized parasites develop into trophozoites, which further asexually divide into merozoites. Merozoites are then released by rupture of the host red blood cells and invade healthy erythrocytes. **Gamogony**. Sexual multiplication of the parasite starts by gametocytes appearing in the host red blood cells. During blood uptake by ticks, gametocytes develop into gametes that mature in the tick midgut lumen. Gamete fertilization then gives rise to a zygote that penetrates the tick peritrophic matrix to tick epithelial cells. Inside these, the zygote undergoes a meiotic division and results in the formation of kinetes, which are released to the haemolymph. The kinetes of *Theileria* species directly invade salivary glands (primary kinetes) but kinetes of *Babesia* parasites are subjected to two series of asexual multiplication in various tick tissues and subsequent secondary kinetes invade the tick salivary glands. **Sporogony**. Sporogony starts after kinete invasion of tick salivary glands, which form the sporont, a polymorphous syncytium. The sporont later evolves into a multinucleated meshwork referred as a sporoblast, which is dormant during tick ecdysis. Maturation of the parasite sporoblast starts after tick attachment to the host and results in sporozoites being released into tick saliva.

### Schizogony

Intra-leukocytic asexual reproduction (Figure 1A) occurs in the lifecycle of two evolutionary lineages, *Theileria* sensu stricto (including *Cytauxzoon* spp.) and its sister clade, represented by *Theileria equi* (Table 1) (Kappmeyer et al., 2012; Schnittger et al., 2012; Schreeg et al., 2016). Schizogony serves to aid rapid parasite multiplication and gives rise to schizonts, referred to as Koch's bodies (Mehlhorn and Shein, 1984; Mehlhorn and Schein, 1993). These *Theileria* intra-leukocytic schizonts are able to modulate the host's immune response, e.g., to block host cell apoptosis (Blouin et al., 1987; Kawai et al., 1989; Sato et al., 1994; Hagiwara et al., 1997; Ahmed et al., 1999; Dobbelaere and Heussler, 1999; Susta et al., 2009). Moreover, leukocyte infection by *Theileria* parasites could lead to a fundamental change in the infected host cell's ability to proliferate indefinitely (Mehlhorn and Shein, 1984; Mehlhorn and Schein, 1993; Ahmed et al., 1999; Dobbelaere and Heussler, 1999; Dobbelaere and Rottenberg, 2003). Although schizogony in nucleated blood cells characterizes all *Theileria* parasites, the neoplastic transformation of the host cell was reported only for *Theileria parva, Theileria annulata, Theileria lestoquardi, Theileria taurotragi*, and *Theileria* sp. (buffalo) (Table 1; Ahmed et al., 1999; Dobbelaere and Heussler, 1999; Dobbelaere and Küenzi, 2004; Zweygarth et al., 2009; Sivakumar et al., 2014; Bishop et al., 2015). Changes in the host cell have not been described for *T. equi* (Schein et al., 1981; Moltmann et al., 1983a; Ramsay et al., 2013), presumably due to the absence of homologs of the putative *Theileria* host cell transforming genes (Kappmeyer et al., 2012; Schreeg et al., 2016).

The sporozoite invasion into nucleated blood cells is a complex process requiring numerous alterations in the metabolism of invading sporozoites (Shaw, 1995, 1996a,b), complicity of the host system (Shaw et al., 1991, 1993; Shaw, 1996a,b) and the involvement of tick saliva (Shaw et al., 1993). In contrast to other apicomplexan parasites, including *Babesia, Theileria* sporozoites are immotile (Table 1; Shaw, 1999, 2003). The initial contact of the parasite and host cell membrane thus occurs randomly (Shaw, 2003). Sporozoite attachment and internalization into the host cell does not require apical-end first orientation and the parasite enters in any orientation (Shaw, 1999, 2003). Thus, the proteins excreted by apical organelles are more involved in the establishment in the host cell cytoplasm rather than the entry process (Shaw et al., 1991). The invasion process comprises several consecutive stages and is completed within about 3 min: (i) recognition and attachment to the host cell membrane; (ii) formation of junctions between the parasite and the host cell membrane; (iii) a “zippering” process resulting in fully internalized parasite in the host cell, yet still surrounded by the host cell membrane; (iv–v) separation and progressive dissolution of the enclosing host cell membrane; and (vi) appearance of a microtubule network derived from the host cell and closely associated with the developing parasite (Fawcett et al., 1982c, 1984; Shaw et al., 1991; Shaw, 1997, 2003). During the invasion process the sporozoite sheds its coat (Dobbelaere et al., 1985; Webster et al., 1985; Shaw, 2003) and lies loosely in the host cell cytoplasm once it has escaped from the enclosing host membrane; no parasitophorous vacuole is formed (Shaw, 2003).

The internalized *Theileria* sporozoite undergoes a change into a multinucleate schizont (Shaw, 1997, 2003) and schizont-infected cells then circulate in the bloodstream (Dobbelaere and Heussler, 1999). The *Theileria* schizogony is accompanied by a series of ultrastructural changes affecting internal organelles as well as the outer surface of the parasite (Shaw and Tilney, 1992). The intra-leukocytic schizogony ends with the production of uninucleated merozoites released into the host bloodstream where they invade erythrocytes (Shaw and Tilney, 1992). The process of *Theileria* merozoite internalization into erythrocytes occurs in the same manner as sporozoite invasion of leukocytes (Shaw and Tilney, 1995). All *Theileria* parasites reproduce in erythrocytes but the process has been described in only a limited number of species (Conrad et al., 1986; Bishop et al., 2004).

### Merogony

Exclusive intra-erythrocytic multiplication (Figures 1B,C) represents the cognitive feature of *Babesia* parasites but the absence of schizogony has not yet been demonstrated for several species of all three evolutionary distinct *Babesia* lineages, *Babesia* sensu stricto, Western *Babesia* group, and *Babesia microti* group (Table 1; Rudzinska and Trager, 1977; Kjemtrup et al., 2006; Lobo et al., 2012; Schreeg et al., 2016). The first contact between *Babesia* invasive stages and the host cell occurs through several random collisions. Unlike *Theileria, Babesia's* orientation of the apical (anterior) end establishes the junction between parasite and host cell membrane (Rudzinska et al., 1976; Montero et al., 2009; Asada et al., 2012; Lobo et al., 2012). Parasite orientation and penetration is mediated by proteins secreted from the apical secretory organelles (Dubremetz et al., 1998; Soldati et al., 2004), and thus is accompanied by apposition of apical organelles with the host cell membrane (Ward and Jack, 1981). Similarly with *Theileria, Babesia* internalizes within a few minutes without parasitophorous vacuole formation (Table 1), and thus the parasite lies freely within the host cell cytoplasm (Simpson et al., 1963; Rudzinska et al., 1976; Potgieter and Els, 1977b; Kawai et al., 1999a,b; Guimarães et al., 2003; Montero et al., 2009; Sun et al., 2011; Lobo et al., 2012). The invasion process does not differ for *Babesia* sensu stricto species (Montero et al., 2009; Asada et al., 2012) and *B. microti* (Rudzinska, 1976; Rudzinska et al., 1976). The internalized *Babesia* sporozoites develop into trophozoites (also described as ring stages), which further asexually divide and produce merozoites by a process referred to as merogony (Box 1, Figure 1) (Rudzinska and Trager, 1977; Fawcett et al., 1987; Montero et al., 2009; Lobo et al., 2012). Later, merozoites are released from ruptured cells and invade other intact and healthy erythrocytes. The merogony of piroplasms is asynchronous (Table 1), and thus trophozoites and merozoites occur in the bloodstream simultaneously (Jalovecka et al., 2016). The short-time residence of the parasite outside the host cell is characterized by the appearance of a fuzzy coat created from fibrillary material and hypervariable surface proteins. The coat occurs on the surface of both *Theileria* and *Babesia* free merozoites and is cut off during invasion of the host cell (Rudzinska et al., 1976; Shaw, 2003; Montero et al., 2009).

The size of merozoites varies according to the piroplasm species as well as the vertebrate host species. The merozoites of piroplasms are characterized by a piriform shape, forming pairs or tetrads (Potgieter and Els, 1977a,b; Lewis et al., 1980; Mehlhorn and Shein, 1984; Conrad et al., 1985, 1986; Kawai et al., 1986, 1999b; Gorenflot et al., 1991, 1992; Shaw and Tilney, 1995; Shaw, 2003; Wise et al., 2013; Del Carmen Terrón et al., 2016). However, divergently shaped merozoites were recorded for species of two early divergent lineages of piroplasms, the Western *Babesia* clade and the *Babesia microti* clade (Table 1) (Rudzinska, 1976; Kjemtrup et al., 2006; Clancey et al., 2010). They possess smaller merozoites of ovoid shape, which later become polymorphic and form numerous invaginations and pseudopods, twisting and coiling. Although all piroplasms exhibit a much reduced apical complex (e.g., absence of conoid) compared to the other apicomplexan parasites (Votypka et al., 2017), *B. microti* merozoites apical complex displays only a single large rhoptry and lack polar rings and a microtubular section (Rudzinska, 1976). The question of feeding mechanisms of piroplasms has not yet been answered. There is a general consensus that piroplasms phagocytose or pinocytose the host cytoplasm (Rudzinska and Trager, 1962; Conrad et al., 1985; Fawcett et al., 1987; Guimarães et al., 2003), but food vacuoles full of host cytoplasm were observed in both *Theileria* and *Babesia* merozoites, suggesting potential extracellular digestion of host cytoplasm (Rudzinska and Trager, 1962; Simpson et al., 1963; Rudzinska, 1976; Rudzinska et al., 1976; Simpson and Neal, 1980; Conrad et al., 1985; Fawcett et al., 1987; Guimarães et al., 2003). If piroplasms can directly digest host hemoglobin still remains a question; this phenomenon was so far suggested only for some *Theileria* species but potential host hemoglobin digestion is not accompanied by pigment or other visible residues formation (Votypka et al., 2017).

## Gamogony: sexual reproduction in the gut of the tick vector

The first sexual stages of piroplasms are referred to as gametocytes (misinterpreted as gamonts in older studies) and appear in the host red blood cells (Box 1, Figure 1, Table 1) (Rudzinska et al., 1979; Mehlhorn and Shein, 1984; Mackenstedt et al., 1990; MacKenstedt et al., 1995; Mehlhorn and Schein, 1993; Gauer et al., 1995; Becker et al., 2010; Bastos et al., 2013; Jalovecka et al., 2016). Gametocytes are predetermined to further differentiate into gametes in the lumen of the tick gut (Rudzinska et al., 1979; Bishop et al., 2004; Becker et al., 2010, 2013), and thus mediate the ability to infect the tick vector (Uilenberg, 2006; Lobo et al., 2012; Becker et al., 2013). Unlike the normally growing and asexually reproducing merozoites, the gametocytes do not reproduce (Rudzinska et al., 1979; Mackenstedt et al., 1990; MacKenstedt et al., 1995; Lobo et al., 2012). They are believed to be larger and unusually shaped compared to asexual stages, however, light microscopy does not allow their reliable recognition (Rudzinska et al., 1979; Lobo et al., 2012). As documented for *B. microti* by electron microscopy, intra-erythrocytic gametocytes are characterized by undifferentiated cytoplasm, large nuclei and unusually twisted, convoluted or folded shapes (Rudzinska et al., 1979). The gametocytes persistence in the host bloodstream was documented for many species of *Babesia* sensu stricto (Mackenstedt et al., 1990; MacKenstedt et al., 1995; Becker et al., 2010, 2013; Bastos et al., 2013; Jalovecka et al., 2016) and *B. microti* (Rudzinska et al., 1979, 1983b). It is generally assumed that sexual commitment of *Theileria* is identical to *Babesia* and gametocytes occur in the circulating blood (Mehlhorn and Shein, 1984; Mehlhorn and Schein, 1993; Zapf and Schein, 1994b; Gauer et al., 1995; Shaw, 2003; Bishop et al., 2004; Uilenberg, 2006). This is supported by recent descriptions of genes with expression specific to sexual commitment in intra-erythrocytic stages of *Theileria* sensu stricto species (Pieszko et al., 2015; Lempereur et al., 2017) and *T. equi* (Bastos et al., 2013). The gametocytes are taken up into the tick midgut together with the blood meal. Subsequently, ingested asexual intra-erythrocytic stages are rapidly destroyed in the gut lumen (Rudzinska et al., 1979; Mehlhorn and Shein, 1984; Bishop et al., 2004; Lobo et al., 2012). Still hidden inside the red blood cells, gametocytes of both *Theileria* and *Babesia* parasites start reorganizing the cytoplasm (Schein et al., 1977; Rudzinska et al., 1979; Mehlhorn et al., 1980; Zapf and Schein, 1994b). Gametocyte metamorphosis is asynchronous, presumably due to non-contemporary blood uptake (Rudzinska et al., 1984). The process is accompanied by microtubular reorganization and gametocytes became completely stretched out compared to previously folded intra-erythrocytic forms (Friedhoff and Büscher, 1976; Weber and Friedhoff, 1977; Rudzinska et al., 1979; Mehlhorn and Shein, 1984; Zapf and Schein, 1994b). As documented for *B. microti*, development of the gametocytes is completed outside of the already lysed erythrocytes in the lumen of the midgut (Rudzinska et al., 1979). Yet, in some cases the process can be completed inside the erythrocyte in the environment of the tick lumen (Rudzinska et al., 1984; Gough et al., 1998).

Metamorphosis of the gametocytes results in the formation of gametes (Figure 1), referred to as Strahlenkörper or spiky-rayed stages (Table 1) (Mehlhorn and Schein, 1993). It was suggested that gametes multiply to form large aggregates but once division is completed, haploid gametes are released to the tick midgut lumen (Warnecke et al., 1980; Mehlhorn and Schein, 1993; MacKenstedt et al., 1995; Gough et al., 1998; Bock et al., 2004). The appearance of piroplasm gametes is unique among apicomplexan parasites and characteristic structures—tail, arms, and arrowhead—begin to form in gametocytes (Rudzinska et al., 1984). Gametes of both *Theileria* and *Babesia* species are haploid (Mackenstedt et al., 1990; MacKenstedt et al., 1995; Gauer et al., 1995) and are considered to be anisogametes, although *Babesia* gametes appear as isogametes when examined by light microscopy (Mehlhorn and Shein, 1984; Gough et al., 1998). The gametes of *Theileria* sensu stricto and *T. equi* are clearly distinguishable by light microscopy as micro- and macro-gametes (Table 1). The characteristic ray bodies are considered to be micro-gametes and macro-gametes are spherically shaped without protrusions (Schein et al., 1977; Warnecke et al., 1980; Mehlhorn and Shein, 1984; Mehlhorn and Schein, 1993; Zapf and Schein, 1994b; Bishop et al., 2004; Uilenberg, 2006). Gametes of *Babesia* sensu stricto species as well as *B. microti* do not differentiate into macro- and micro-gametes but two gamete populations are formed (Table 1). These two types differ in the details of cytoplasm density and shape (Friedhoff and Büscher, 1976; Rudzinska et al., 1979, 1983b; Mehlhorn et al., 1981; Gough et al., 1998).

Fertilization of piroplasms is induced by close contact between two gametes of different types and may occur at very early stages of gamete formation (Rudzinska et al., 1983b). Filamentous structures are formed between membranes of closely adjacent gametes. Subsequently, a finger-like protrusions of one gamete penetrates the opposite one (Mehlhorn et al., 1981; Rudzinska et al., 1983b; Mehlhorn and Shein, 1984). Gamete fertilization results in the formation of a zygote (Schein et al., 1977; Mehlhorn et al., 1979; Warnecke et al., 1980; Rudzinska et al., 1983b, 1984; Mackenstedt et al., 1990; MacKenstedt et al., 1995; Higuchi et al., 1991a, 1999a,b; Zapf and Schein, 1994b; Gauer et al., 1995; Gough et al., 1998; Bishop et al., 2004). The zygote of Piroplasmida is a motile stage that is often referred to as an ookinete or kinete. However, such nomenclature is misleading since kinetes (often also called ookinetes or sporokinetes) represent haploid stages resulting from the meiotic division of a diploid zygote (Mehlhorn et al., 1978, 1979; Mehlhorn and Shein, 1984; Rudzinska et al., 1984). To further develop, the zygote penetrates the peritrophic matrix (Figure 1, Table 1) (Rudzinska et al., 1982), which appears temporarily during feeding at all tick stages and compartments the gut lumen into endo-peritrophic and ecto-peritrophic spaces (Sonenshine, 1991). Since the peritrophic matrix represents a strong mechanical barrier, zygote penetration is an active process accomplished by enzymes released from the arrowhead structure of the zygote (Rudzinska et al., 1982, 1984). Matrix penetration starts immediately after zygote formation (Rudzinska et al., 1982, 1983a,b). The arrowhead structure opens the way for the zygote body by release of enzymes. Subsequently, the zygote enters the ecto-peritrophic space and immediately invades gut epithelial cells. This cell invasion is triggered by the arrowhead structure of the zygote but the arrowhead does not pierce the cell membrane; the membrane remains intact. Once the zygote is internalized into the epithelial cell, the invagination membrane disappears. Thus, the zygote occurs loosely in the cytoplasm of the epithelial cell and is surrounded by cell organelles. Inside the epithelial cell, the zygote turns into a spherical shape. Simultaneously, the arrowhead structure loses its organized pattern and gradually disappears (Rudzinska et al., 1982, 1983a). Zygote penetration of the peritrophic matrix and internalization into epithelial cells has been described in detail only for *B. microti* species but is generally assumed to be consistent for all Piroplasmida (Mehlhorn and Schein, 1993). Once morphological changes are finalized, the zygote undergoes a meiotic division as evidenced by DNA measurements of *Theileria* and *Babesia* species (Gauer et al., 1995; MacKenstedt et al., 1995). Meiosis inside the epithelial cell results in the formation of kinetes as was documented for species of both *Theileria* sensu stricto and *Babesia* sensu stricto lineages, as well as for *T. equi* and *B. microti* (Table 1) (Potgieter et al., 1976; Potgieter and Els, 1977a; Mehlhorn et al., 1978; Warnecke et al., 1980; Rudzinska et al., 1984; Zapf and Schein, 1994b).

The kinetes are released from the gut epithelial cells into the tick haemolymph (Figure 1) (Potgieter et al., 1976; Potgieter and Els, 1977a; Mehlhorn et al., 1978, 1979; Schein and Friedhoff, 1978; Warnecke et al., 1980; Mehlhorn and Shein, 1984; Rudzinska et al., 1984; Karakashian et al., 1986; Higuchi et al., 1991b; Zapf and Schein, 1994b), where the motile kinetes are disseminated via the haemolymph throughout the whole tick body and invade internal tissues. Kinetes are primarily uni-nucleated but exceptionally, kinetes with more nuclei can occur in the haemolymph due to the early beginning of nuclear division (Mehlhorn and Schein, 1993). In the haemolymph, as other invasive stages of piroplasms, kinetes are covered with a fuzzy coat created from fibrillary material and hypervariable surface proteins (Karakashian et al., 1986). The kinetes of *Babesia* sensu stricto species are subjected to two cycles of asexual multiplication (Table 1) (Potgieter and Els, 1977a; Mehlhorn et al., 1980; Mehlhorn and Shein, 1984; Mehlhorn and Schein, 1998). In the first, the *Babesia* kinetes invade various tick tissues like haemocytes, muscle fibers, Malpighian tubules, peritracheal cells, and ovaries of adult females. Here, the kinetes undergo the second asexual multiplication (Potgieter and Els, 1977a; Moltmann et al., 1982b; Mehlhorn and Shein, 1984). Subsequently, the secondary kinetes invade the salivary glands where sporogony, the maturation of sporozoites, takes place (Christophers, 1907; Friedhoff et al., 1972; Potgieter and Els, 1976; Weber and Friedhoff, 1979; Moltmann et al., 1982a; Mosqueda et al., 2004). Species of the *Babesia* sensu stricto lineage possess a unique feature among all apicomplexan parasites; transovarial transmission (Figure 1, Table 1). This process is mediated by *Babesia* invasion into the ovarian cells and transmission via larval progeny to tick larvae (Joyner et al., 1963; Donnelly and Peirce, 1975; Lewis and Young, 1980; Moltmann et al., 1982b; Mehlhorn and Shein, 1984; Higuchi et al., 1993; Mehlhorn and Schein, 1993; Bonnet et al., 2007; Boldbaatar et al., 2008, 2010). No transovarial transmission occurs in the lifecycle of *B. microti* or *Theileria* species (Table 1). The kinetes of *B. microti* primarily invade fat body (nephrocytes) and salivary glands. Inside, the kinetes form the kinetoblast, which differentiates to produce secondary kinetes. Subsequently, secondary kinetes invade salivary glands to undergo sporogony (Karakashian et al., 1986). Kinetes of *Theileria* sensu stricto species and *T. equi* are believed to migrate directly to salivary glands (Table 1) as no kinete invasion of other tick tissues has been documented (Mehlhorn et al., 1979; Moltmann et al., 1983b; Mehlhorn and Shein, 1984; Mehlhorn and Schein, 1993, 1998; Zapf and Schein, 1994a; Uilenberg, 2006).

Remarkable differences have been documented in size and chronological order of piroplasm sexual development. Such divergence can be attributed to the variety of the piroplasm species and the wide spectrum of both vertebrate hosts and vectors. Gametes of *Babesia* parasites develop during tick feeding, appear before full tick engorgement and within ~3 days post tick repletion. Subsequently, the kinetes are found in the tick haemolymph from ~2 to ~6 days post repletion. *Theileria* sexual development seems to be a longer process; the first appearance of gametes was documented between ~1 and ~5 days post tick repletion and kinetes released to the haemolymph were first seen from ~13 to ~34 days post repletion. In general, the length of piroplasm sexual development correlates with the feeding duration of tick developmental stages (larvae vs. nymphs vs. adults), and tick developmental differences derived from the number of host species (one- vs. two- vs. three-host ticks) (Friedhoff and Büscher, 1976; Potgieter et al., 1976; Potgieter and Els, 1977a; Schein et al., 1977; Weber and Friedhoff, 1977; Mehlhorn et al., 1978, 1979, 1980; Warnecke et al., 1980; Rudzinska et al., 1982, 1983a,b, 1984; Mehlhorn and Shein, 1984; Mackenstedt et al., 1990; MacKenstedt et al., 1995; Higuchi et al., 1991a, 1992, 1999a,b; Mehlhorn and Schein, 1993; Zapf and Schein, 1994b; Gauer et al., 1995; Gough et al., 1998).

## Sporogony: asexual reproduction in the salivary gland of the tick vector

The kinetes of Piroplasmida parasites further develop in tick salivary glands to produce invasive stages referred to as sporozoites (Figure 1). The sporozoites mediate parasite transmission from the tick vector to the vertebrate host. Piroplasms develop in acini of types II and III (Fawcett et al., 1982a,b), which represent the majority of acini in the typical grape-like structure of salivary glands (Coons and Roshdy, 1973; Binnington, 1978). Sporogony (Box 1) begins with invasion of tick salivary glands by piroplasm kinetes (Christophers, 1907; Holbrook et al., 1968; Purnell and Joyner, 1968; Friedhoff et al., 1972; Potgieter and Els, 1976; Schein et al., 1979; Weber and Friedhoff, 1979; Weber and Walter, 1980; Fawcett et al., 1982a,b; Moltmann et al., 1982a, 1983b; Karakashian et al., 1983; Blouin and van Rensburg, 1988; Blouin and De Waal, 1989; Higuchi et al., 1994; Zapf and Schein, 1994a; Guimarães et al., 1998a,b). Invading kinetes rapidly enlarge and transform into the polymorphous single-membrane syncytium referred to as a sporont. Later, the sporont evolves into a sporoblast, a multinucleated and relatively undifferentiated three-dimensional branching meshwork which has already developed before the beginning of tick ecdysis and molting. The formation of a sporoblast is associated with hypertrophy of the infected acini cells (Table 1), which is a common feature of sporogony in *Theileria* sensu stricto species (Purnell and Joyner, 1968; Schein and Friedhoff, 1978; Mehlhorn et al., 1979; Fawcett et al., 1982a,b), *T. equi* (Zapf and Schein, 1994a) and *B. microti* (Weber and Walter, 1980; Karakashian et al., 1983; Piesman et al., 1986; Yano et al., 2005). The same phenomena were documented for some *Babesia* sensu stricto species like *B. bovis* (Potgieter and Els, 1976) and *B. canis* (Schein et al., 1979) but not for *B. ovis* (Friedhoff et al., 1972; Moltmann et al., 1982a) or *B. caballi* (Blouin and De Waal, 1989). During tick ecdysis, the sporoblast appears to be dormant and its maturation starts when the molted tick attaches to the host. However, a unique formation of fully matured sporozoites prior to tick molting was documented for *T. equi* (Table 1). Sporozoites developed during parasite acquisition and were competent to transmit and expand the infection to the naïve host. Later, the secondary sporozoites developed after attachment of the molted tick stages (Zapf and Schein, 1994a).

Sporoblast maturation of *Babesia* sensu stricto species begins with the appearance of numerous cytomeres (Table 1) (Friedhoff et al., 1972; Potgieter and Els, 1977a; Schein et al., 1979; Moltmann et al., 1982a) but cytomere formation was absent in *Theileria* species (Fawcett et al., 1982a,b; Hazen-Karr et al., 1987) and *B. microti* (Karakashian et al., 1983). The structures of the apical complex appear subsequently, but prior to the sporozoites budding off the sporoblast (Karakashian et al., 1983). Some skeletal components of the apical complex—the conoid and microtubules—are absent in sporozoites of all piroplasms (Friedhoff et al., 1972; Schein and Friedhoff, 1978; Mehlhorn et al., 1979; Schein et al., 1979; Fawcett et al., 1982a,b; Karakashian et al., 1983; Moltmann et al., 1983b; Piesman et al., 1986; Zapf and Schein, 1994a). Polar rings are formed in sporozoites of *Babesia* sensu stricto species (Table 1) (Friedhoff et al., 1972; Schein et al., 1979; Weber and Friedhoff, 1979; Moltmann et al., 1982a; Blouin and van Rensburg, 1988; Blouin and De Waal, 1989) and *T. equi* (Moltmann et al., 1983b) but do not appear in sporozoites of *B. microti* (Karakashian et al., 1983; Yano et al., 2005) and *Theileria* sensu stricto species (Fawcett et al., 1982a,b). Thus, *B. microti* sporogony is more reminiscent of *Theileria* sensu stricto species with respect to sporoblast structure. The formation of sporozoites is attributed to the process of multiple fissions, referred to as budding (Table 1). Apart from *B. canis*, where sporozoite differentiation was described as a result of successive binary fissions (Schein et al., 1979), the budding process was documented for both *Theileria* and *Babesia* species (Holbrook et al., 1968; Friedhoff et al., 1972; Potgieter and Els, 1976; Schein and Friedhoff, 1978; Fawcett et al., 1982a; Moltmann et al., 1982a, 1983b; Hazen-Karr et al., 1987; Blouin and van Rensburg, 1988; Blouin and De Waal, 1989). Since the parasitophorous vacuole is not formed, the sporogony stages are in immediate contact with the host-cell cytoplasm (Friedhoff et al., 1972; Fawcett et al., 1982a; Moltmann et al., 1982a, 1983b). Piroplasm sporogony is asynchronous and the various developmental stages occur within individual acinar cells (Karakashian et al., 1983; Blouin and van Rensburg, 1988; Yano et al., 2005). This is attributed to the continuous release of sporozoites into the tick saliva and to the bloodstream of the vertebrate host during the several days of tick feeding (Yano et al., 2005). Sporogony ends with longish piriform sporozoites equipped with apical organelles that later mediate internalization to the host blood cell.

Sporogenic events and progress are assumed to be consistent for both *Babesia* and *Theileria* species (Christophers, 1907; Purnell and Joyner, 1968; Friedhoff et al., 1972; Potgieter and Els, 1976; Weber and Friedhoff, 1979; Fawcett et al., 1982a; Moltmann et al., 1982a, 1983b; Karakashian et al., 1983; Higuchi et al., 1994; Zapf and Schein, 1994a; Guimarães et al., 1998a,b; Mehlhorn and Schein, 1998; Mosqueda et al., 2004) but alteration in the process length and sporozoite size occurs. Such a discrepancy is attributed to the variety of tick species and their natural developmental characteristics such as the number of host species (one- vs. two- vs. three-host ticks). Generally, sporozoite maturation after tick attachment to the host lasts at least 48 h, as documented for representatives of lineages *Babesia* sensu stricto, *Theileria* sensu stricto, *T. equi* and *B. microti* (Mehlhorn et al., 1979; Karakashian et al., 1983; Takahashi et al., 1993; MacKenstedt et al., 1995; Guimarães et al., 1998a,b).

## Conclusion and future perspectives: the role of the life cycle in anti-piroplasm strategies

Species of the group Piroplasmida possess a characteristic lifecycle that significantly differs from other apicomplexan parasites. A uniform consensus describes three consecutive phases (Figure 1): (i) schizogony and merogony, asexual multiplication in blood cells of the vertebrate host; (ii) gamogony, sexual reproduction inside the tick midgut, later followed by kinete invasion of the tick internal tissues; and (iii) sporogony, asexual proliferation in tick salivary glands resulting in the formation of sporozoites (Box 1). However, the order Piroplasmida includes many species spread into five evolutionary distinct lineages. Thus, in the lifecycles of piroplasms many fundamental variations occur from the general consensus and these discrepancies need to be taken into account in the development of anti-piroplasm strategies.

To date, the majority of inventions in anti-piroplasm vaccine development exploits the vertebrate host stages: schizonts and/or merozoites (Florin-Christensen et al., 2014; Nene and Morrison, 2016). Trends in anti-babesial strategies operate particularly with subunit vaccines based on merozoite surface antigens since recently, many of these have been characterized (recently reviewed in Florin-Christensen et al., 2014). In general, these surface antigens exhibit high immunogenicity and antibodies against them are able to mediate inhibition of parasite intra-erythrocytic invasion and development (Florin-Christensen et al., 2014). Analogously, surface antigens of schizont-infected cells represent hot candidates for anti-theilerial vaccines (Nene and Morrison, 2016). Sporozoites, crucial piroplasm stages responsible for parasite transmission from the tick vector to the vertebrate host, are currently the center of interest for anti-piroplasm strategies (Florin-Christensen et al., 2014; Nene and Morrison, 2016; Nene et al., 2016) but this research is restricted by the absence of effective laboratory transmission models, particularly for species in lineages of *T. equi* and *B. microti*. So far, a few sporozoite surface antigens have been defined but they generally displayed a low level of immunogenicity (Florin-Christensen et al., 2014; Nene and Morrison, 2016; Nene et al., 2016). However, antibodies neutralizing sporozoite infectivity have been demonstrated in animals exposed to repeated sporozoite challenges (Nene and Morrison, 2016). On the contrary, very limited knowledge of intra-tick developmental stages restricts research on tick-pathogen interactions. Up to now, only few vaccine candidates were defined for species, particularly of the *Babesia* sensu stricto lineage, and partially also of the *B. microti* group (Hajdušek et al., 2013; de la Fuente et al., 2017) but there is lack of knowledge about *Theileria* and tick interactions. Although intra-tick development differs among all piroplasm evolutionary lineages, comparative bioinformatics analysis implies a high level of conservation of crucial regulatory domains responsible for piroplasm life cycle transitions (Alzan et al., 2016). Targeting key *Babesia* or *Theileria* developmental stages in tick tissues represents an attractive way toward transmission-blocking vaccines. However, this research requires in depth knowledge of parasite intra-tick development with a strong focus on conserved or divergent developmental features.

## Author contributions

MJ, OH, and LM conceived and designed the review. DS and PK contributed ideas and concepts. MJ did the literature search, wrote the manuscript, and designed the figure. MJ, OH, DS, PK, and LM finalized the paper and figure.

### Conflict of interest statement

The authors declare that the research was conducted in the absence of any commercial or financial relationships that could be construed as a potential conflict of interest. The reviewer JL declared a shared affiliation, with no collaboration, with several of the authors, MJ, OH, DS, and PF, to the handling Editor.
